# Supplementary material for: Assessing the Benefits and Costs of the Hydrogen Cyanide Antiherbivore Defense in Trifolium repens
Source: Plants (Basel). 2023 Mar 7;12(6):1213. doi: 10.3390/plants12061213 (PMC10056272; doi:10.3390/plants12061213)
Supplement: Supplementary file 1 [file plants-12-01213-s001.zip › plants-2257480-supplementary.pdf]

Supplementary tables and figures

Assessing the Benefits and Costs of the Hydrogen Cyanide Antiherbivore Defense in *Trifolium repens*

Hind Emad Fadoul [1,*], Lucas J. Albano [1,2], Matthew E. Bergman [1,3], Michael A. Phillips [1,3], Marc T. J. Johnson [1,2,*]

1 Department of Biology, University of Toronto Mississauga, Mississauga, ON L5L 1C6, Canada
2 Department of Ecology and Evolutionary Biology, University of Toronto, Toronto, ON M5S 3B2, Canada
3 Department of Cell and Systems Biology, University of Toronto, Toronto, ON M5S 3G5, Canada
* Correspondence: hind.emad@utoronto.ca (H.E.F.); marc.johnson@utoronto.ca (M.T.J.J.)

Table S1: Results from linear models for herbivory from no-choice assay trial 1 with all zeroes (no herbivory) excluded. Data were square-root transformed.

Effect	F	d.f.	P
Cyanotype	6.282	3	**< 0.001**
Temperature	6.560	1	**0.013**
Cyanotype × Temperature	3.852	3	**0.014**

* For each response variable, we tested the effects of leaf cyanotype, temperature (15°C and 25°C), and the interaction between cyanotype and temperature. For each effect, we show the degrees-of-freedom (d.f.), F- and P-value; the degrees of freedom for the error term in all assays was 56. Significant differences (P < 0.05) are indicated in **bold**.

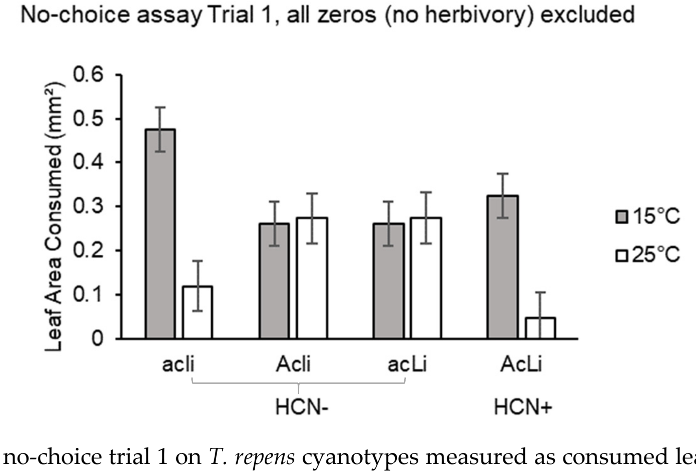

Figure S1: Slug herbivory from no-choice trial 1 on *T. repens* cyanotypes measured as consumed leaf area (mm^2) with all zeroes (no herbivory) excluded at two different temperatures (N = 10 plants per cyanotype at each temperature) over a 24h period. Bars represent means ± SE.

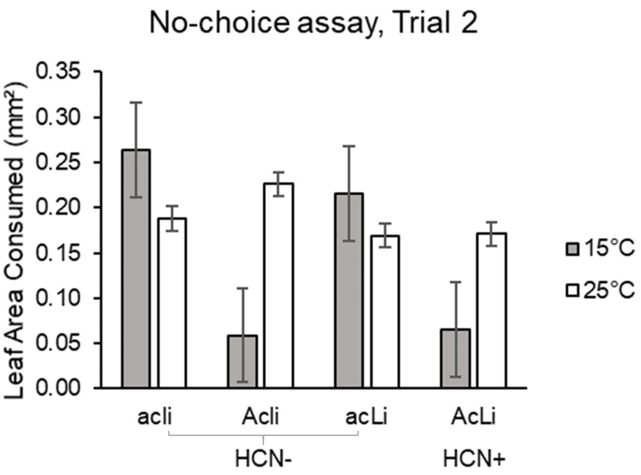

Figure S2: Slug herbivory from no-choice trial 2 on *T. repens* cyanotypes measured as consumed leaf area (mm^2) at two different temperatures (N = 10 plants per cyanotype at each temperature) over a 24h period. Bars represent means ± SE

Table S2: Results from linear models for herbivory from no-choice assay trial 2 with all zeroes (no herbivory) excluded.

Effect	F	d.f.	P
Cyanotype	0.721	3	0.546
Temperature	0.071	1	0.792
Cyanotype × Temperature	0.810	3	0.497

* For each response variable, we tested the effects of leaf cyanotype, temperature (15°C and 25°C), and the interaction between cyanotype and temperature. For each effect, we show the degrees-of-freedom (d.f.), F- and P-value; the degrees-of-freedom for the error term was 34.

Table S3: Results from linear models for herbivory from choice assay with all zeroes (no herbivory) excluded.

Effect	F	d.f.	P
Cyanotype	0.041	3	0.988
Temperature	1.043	1	0.319
Cyanotype × Temperature	0.237	3	0.869

* For each response variable, we tested the effects of leaf cyanotype, temperature (15°C and 25°C), and the interaction between cyanotype and temperature. For each effect, we show the degrees-of-freedom (d.f.), F- and P-value; the degrees of freedom for the error term was 20.

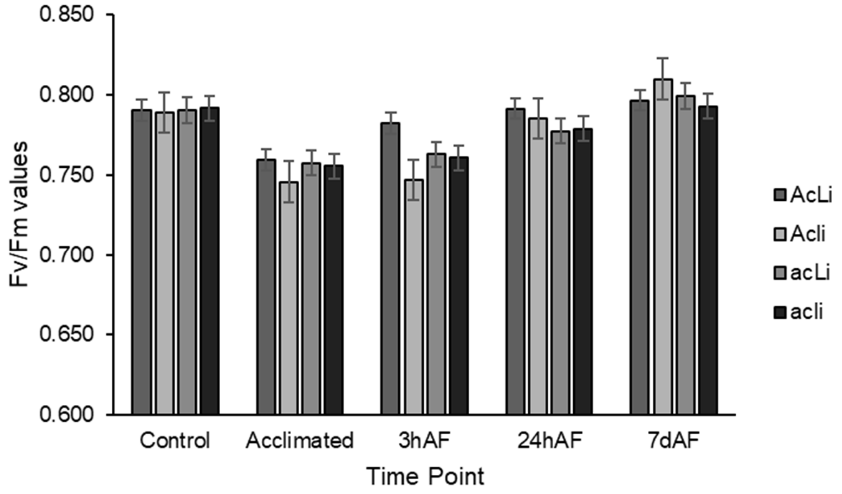

Figure S3: Fv/Fm in four *T. repens* cyanotypes at four time points; Control (no freezing), 3h after freezing (3hAF), 24h after freezing (24hAF) and 7d after freezing (7dAF).
